# Long-Term Training Increases Atrial Fibrillation Sustainability in Standardbred Racehorses

**DOI:** 10.1007/s12265-023-10378-6

**Published:** 2023-04-04

**Authors:** Helena Carstensen, Sarah Dalgas Nissen, Arnela Saljic, Eva Melis Hesselkilde, Arne van Hunnik, Mathias Hohl, Stefan Michael Sattler, Cecilie Fløgstad, Charlotte Hopster-Iversen, Sander Verheule, Michael Böhm, Ulrich Schotten, Thomas Jespersen, Rikke Buhl

**Affiliations:** 1 Department of Veterinary Clinical Sciences, Faculty of Health and Medical Sciences, University of Copenhagen, Højbakkegaard Allé 5, 2630 Taastrup, Denmark; 2 Laboratory of Cardiac Physiology, Department of Biomedical Sciences, Faculty of Health and Medical Sciences, University of Copenhagen, Blegdamsvej 3B, 2200 Copenhagen, Denmark; 3 Department of Physiology, Maastricht University, Maastricht, Netherlands; 4 Department of Internal Medicine III, University Hospital, Saarland University, Homburg, Saar Germany; 5 Department of Cardiology, Herlev and Gentofte University Hospital, Herlev, Denmark

**Keywords:** Exercise, Cardiac remodeling, Athlete’s heart, Atrial fibrillation, Epicardial mapping, Inflammation

## Abstract

Atrial fibrillation (AF) is more prevalent in athletes, and currently, the mechanisms are not fully understood. Atrial fibrillation inducibility and stability was investigated in trained and untrained Standardbred racehorses. The horses underwent echocardiography for evaluation of atrial size. High-density mapping during AF was performed, and the presence of structural remodeling, as well as the expression of inflammatory and pro-inflammatory markers in the atria, was studied. Atrial fibrillation sustained significantly longer after tachypacing in the trained horses, whereas no difference in AF inducibility was found. The untrained horses displayed a significant difference in the AF complexity when comparing right and left atria, whereas such difference was not observed in the trained animals. No evidence of increased structural remodeling or inflammation could be identified. Left atrial dimensions were not significantly increased. The increased AF sustainability in trained horses was not related to fibrosis or inflammation as seen in other animal exercise models.

**Graphical abstract:**

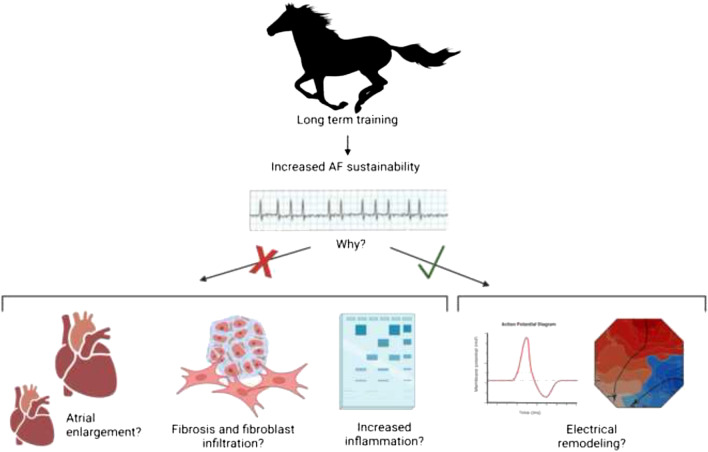

**Supplementary Information:**

The online version contains supplementary material available at 10.1007/s12265-023-10378-6.

## Introduction

For decades, people have been aware of the great benefits of physical training and acknowledged its essential role in maintaining a healthy lifestyle. Furthermore, exercise has become a spectacle that is cultivated by millions of people worldwide and the number of people participating in marathons and triathlons is increasing [1]. However, an increasing concern regarding the potential negative impacts of high-intensity endurance training on cardiac health has emerged [2]. One major concern is the effect of endurance training on the development of cardiac arrhythmias, such as atrial fibrillation (AF) [3–6]. Studies have shown that excessive endurance training is linked to a twofold to fivefold increased risk of developing AF [6, 7]. Despite great efforts, the mechanistic background remains ambiguous. Substantial work delineating the “athlete’s heart” demonstrates that some athletes present cardiac chamber dilation, including left atrial (LA) enlargement [8, 9]. In non-athletes, LA enlargement is a risk factor of AF and AF itself leads to atrial enlargement. However, a direct causal relationship between atrial enlargement in athletes and subsequent AF incidence has not been established. Another commonly presented character of the athlete’s heart is a pronounced change in the nodal activity presented as sinus bradycardia and first-degree atrioventricular (AV) block [10, 11]. In athletes, these conduction abnormalities are considered to be benign features of the trained heart [8, 12–14]; however, bradycardia and prolonged PR interval are also reported to be associated with a higher risk of developing AF [3]. It has been suggested that these conduction changes could be related to an increase in vagal tone [12], which is also known to induce shortening of the atrial effective refractory period (aERP) and to increase the dispersion of the refractory periods in the atria [15]. Atrial refractoriness remains uninvestigated in athletes, and the role of vagal tone in athletes and AF pathophysiology is still an area for further study.

Lately, molecular remodeling has been extensively investigated, revealing that fibrosis and inflammation play a major role in the development of AF, explaining some of the challenges in regard to AF treatment [8, 16]. Whether molecular remodeling occurs in human athletes is still unclear, but studies of murine exercise models undergoing vigorous training showed that several bouts of high-intensity exercise lead to the initiation of inflammatory processes, resulting in the development of fibrosis in the atria [17–19]. However, there is an uncertainty as to what extent these forced training regimens may actually induce a stress response, which, by itself, can cause an increase in the level of inflammation [20, 21]. Whether human athletes develop atrial cardiac fibrosis after long-term training has been investigated. However, the results are contradicting and substantial atrial fibrotic development in humans is unlikely [22–27].

Horses are intensively bred for racing and subjected to demanding training regimens, starting at an early age. Interestingly, trained horses develop cardiac changes equivalent to the human athlete’s heart, including cardiac hypertrophy and exercise-induced electrical remodeling [28–30]. Additionally, some horses, like humans, develop AF where no obvious cause is evident [31]. The prevalence of AF in horses is up to 2.5%, with racehorses being most frequently affected [32, 33]. The racehorse is therefore a relevant animal model when studying both exercise-induced cardiac remodeling and AF vulnerability.

We hypothesized that long-term training causes atrial enlargement and produces electrophysiological and molecular atrial remodeling, leading to an increased AF susceptibility in trained horses.

## Material and Methods

### Animals

Twelve Standardbred racehorses (mean age 6.9 years (range 5–10 years), mean body weight (BW) 485 kg (range 415–565 kg)) were subjected to consistent race training for at least three consecutive years until the start of the protocol and were assigned to the trained group. Overall, the training regimens included intense exercise by riding or driving two to three times per week, along with light exercise such as swim training, walking, or jogging two to three times per week. The horses participated in races one to two times per month on average. Training of the horses continued up until inclusion and further continued at the research facility, where the horses were familiarized with running on a treadmill. The horses were trained every other day at medium intensity (heart rate (HR) ~ 150 beats per min (bpm) for 20 min). During the 3 weeks of inclusion, the horses underwent two to three exercise tests reaching maximum HR. Another twelve Standardbred racehorses (similar in age: mean age 7.1 years (range 4–10 years), *p* = 0.83; BW: mean BW 497 kg (range 404–560 kg), *p* = 0.55) were included as untrained control horses. Out of these, six horses had never been trained before and six horses had previously been trained for approximately 1 year, when they were very young (~ 4 years old). The median duration since they were in training was 4.1 years (IQR: 3.5–4.1 years). These horses were also familiarized with the treadmill; however, training was kept to a minimum, although they also had to be subjected to two to three tests reaching maximum HR. As no difference in left ventricular (LV) mass existed between the untrained and previously trained horses, these were all assigned to the untrained group (Supplemental Table S1). Only horses with no signs of cardiovascular disease were included. However, trivial to mild valvular regurgitations were accepted. Lameness grade < 3 on the American Association of Equine Practitioners lameness grading scale was accepted.

**Fig. 1:**
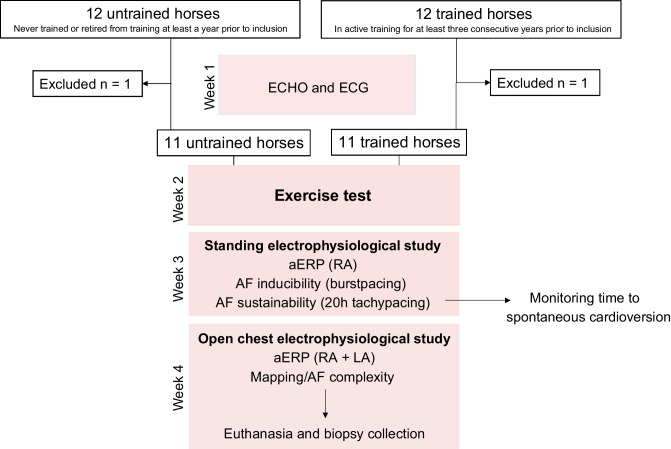
Study outline. Twelve trained and 12 untrained horses were included. After acclimatization, baseline echocardiography (ECHO) and 24-h electrocardiogram (ECG) recordings were obtained. After 2 weeks, the horses were subjected to a standardized exercise test to fatigue. Next, the horses (non-sedated) were included in an electrophysiology (EP) study where atrial effective refractory period (aERP) and atrial fibrillation (AF) inducibility were investigated. The horses were then directly subjected to 20 h of tachypacing. Lastly, the horses were included in a terminal open-chest EP study where epicardial atrial mapping was performed followed by biopsy collection. RA, right atrium; LA, left atrium

All horses were subjected to exercise tests and two electrophysiological (EP) studies. The outline of the study is presented in Fig. 1.

### Performance Evaluation

All horses were subjected to a standardized treadmill exercise test, as previously described [34]. The horses were familiarized with the treadmill (Säto, Knivsta, Sweden) before the test which consisted of an incremental test to fatigue, with initial velocity at 7 m/s which was increased by 0.5 m/s every minute until the horse could no longer keep pace, despite vocal encouragement. An experienced operator blinded to the training status of the horses decided when exhaustion was reached. A base-apex ECG recording was obtained, and blood samples were collected from a catheter placed in the right jugular vein. The fitness level was evaluated according to maximum velocity (*V*_max_), maximum HR (HR_max_), velocity at HR 200 (*V*_200_), maximum blood lactate, and velocity at lactate 4 mmol/l (*V*_LA4_). All horses received flunixin-meglumine (Finadyne® vet, 1.1 mg/kg; MSD, Segré, France) for pain relief 2 h after the test and the following day.

### ECG Analysis

For baseline resting ECG analysis, 10 consecutive beats from the 24-h ECG recordings, where the HR was lowest and where no arrhythmia was present, were chosen and imported to LabChart 8 (v8.1.16, ADInstruments). Heart rate, P wave duration, PR interval, QRS duration, and QT interval were manually measured by a single operator who was kept blinded to the training status. QT interval was corrected for HR using the piecewise linear regression model for Standardbred racehorses [35]. Furthermore, the number of second-degree AV blocks and atrial premature complexes (APCs) were counted from the entire 24-h ECG recording using Televet 100 software version 6.0.0.

### Echocardiography

Echocardiography including two-dimensional, motion mode, and color Doppler was conducted. The following measurements were used for LV mass: left ventricular cavity diameter in diastole (LVIDd), interventricular septum thickness in diastole (IVSd), and LV free wall thickness in diastole (LVFWd) using the following equation: LV_mass_ = 1.04 × ((LVIDd + LVFWd + IVSd)^3^ − LVIDd^3^) − 13.6 that has been validated in horses [36]. Left atrial diameter (LA_Diameter_) was measured during maximal diameter measurement just prior to mitral valve opening as previously described [37]. Left atrial area (LA_Area_) was measured at three different time points: LA_Amax_ = 1 frame before mitral valve opening, LA_Aa_ = at onset of P wave, and LA_Amin_ = at mitral valve closure. LV mass was directly corrected for BW [29], and LA_Diameter_ and LA_Area_ were corrected using allometrical scaling to BW 500 kg using the following equations: LA_Diameter(500)_ = measured LA_Diameter_ / BW^1/3^ × 500^1/3^ and LA_Area(500)_ = measured LA_Area_ / BW^2/3^ × 500^2/3^ [38]. Left atrial function was assessed as previously described [37], including the overall LA fractional area change (LA-FAC_total_ = (LA_Amax_ − LA_Amin_) / LA_Amax_). LA-FAC_passive_ = (LA_Amax_ − LA_Aa_) / LA_Amax_ representing the passive atrial emptying during early diastole and diastasis. LA-FAC_active_ = (LA_Aa_ − LA_Amin_) / LA_Aa_ representing the active atrial contraction.

### Electrophysiological Study (Conscious Horse)

An EP study was performed in the standing, non-sedated horse. An eight-polar electrode (Inquiry Steerable Diagnostic Catheter, 6F/110 cm; St. Jude Medical, Inc., Glostrup, Denmark) was placed through an introducer sheath (Introducer Sheath, Fr. 9.; Terumo Medical Corp., 950 Elkton, MD 21,921, USA) in either the right or left jugular vein and advanced into the right atrium (RA) and finally secured to the horse. The horse was restrained in its stall to prevent excessive moving during the procedure, while still allowing for food and water intake. A base-apex ECG with one lead optimized for atrial activity was recorded during the experiments. The intra-atrial electrode and the surface ECG were connected to an EP system (EP-Workmate™ Recording System V.4.3.2; Abbott Laboratories, MN, USA). Prior to electrophysiological measurements, the threshold for pacing was determined at a pacing cycle length (PCL) of 800 ms. The heart was allowed to adapt to pacing for at least 1 min. Pulse width was 2 ms throughout the procedure, and pacing was performed at 3 × threshold during all procedures.

### Atrial Effective Refractory Period

Atrial effective refractory period (ERP) measurements were performed at a PCL of 1000 ms, 800 ms, 500 ms, and 400 ms. For every 8 stimuli at PCL (S1), an extra stimulus (S2) was applied, increasing with 5 ms each time. The aERP was determined as the longest S1–S2 interval without capture [39]. The aERP was measured three times at each PCL.

### Atrial Fibrillation Susceptibility and Stability

Burst pacing consisted of 200 stimuli with a PCL at 20 ms and amplitude at 10 mA. The burst pacing protocol was repeated up to 20 times or until AF was sustained for > 30 min.

After completion of the EP study, the electrode remained in the RA and tachypacing at a PCL of 100 ms (PW 2 ms, amplitude 10 mA) was initiated and continued for 20 h. During this time, the horse was kept restrained in the stable and monitored continuously to ensure constant atrial capture. After 20 h of tachypacing, the pacing ceased and the horse was released from all equipment except the base-apex ECG device. This was used to monitor for spontaneous cardioversion into sinus rhythm.

### Open-Chest Procedure

All horses were subjected to a terminal open-chest procedure, where high-density (HD) multi-electrode epicardial contact mapping of both atria through left-sided thoracotomy was performed as previously described [40].

The horses were intravenously pre-medicated, sedated, and anesthetized according to previous descriptions [40]. For induction of general anesthesia, zolazepam combined with tiletamine (Zoletil®, 1.5 mg/kg i.v.; Virbac Denmark A/S, Kolding, Denmark) was used and the anesthesia was maintained by isoflurane (IsoFlo Vet., 1.4%; Orion Pharma Animal Health, Copenhagen, Denmark). Prior to anesthesia, two 9-Fr. sheaths (Introducer Sheath, Fr. 9.; Terumo Medical Corp., 950 Elkton, MD 21,921, USA) were placed in the jugular vein for the placement of transvenous electrocardioversion (TVEC) electrodes in the pulmonary artery (PA) and RA for internal defibrillation (Lifepak 20e, Washington, USA) [41] which were also placed prior to induction of general anesthesia. The horse was placed in right lateral recumbency and equipped with a surface ECG. Rib resection of the 5^th^ costae was performed [42] after local analgesia (Carbocain®, 10 mg/ml mepivacaine; Aspen Pharma Trading Limited, Dublin, Ireland), and lastly, the pericardial sac was opened [42].

High-density electrode grids (249 electrodes, 2.5 mm inter-electrode distance) were placed on the atrial epicardium surface to map conduction properties during AF. Mapping consistently started on the left atrium (LA), followed by the RA. Unipolar electrograms were recorded at a sample rate of 1039 Hz, band pass filter (0.1–408 Hz), and 16-bit AD conversion. For evaluation of aERP, a similar pacing protocol as described in the non-sedated horse was used. Most horses spontaneously developed AF, and if not, burst pacing as in the non-sedated horses was used to induce AF. Five minutes of AF was recorded, and sinus rhythm was restored by internal defibrillation if the horse did not spontaneously cardiovert. The same procedure was repeated on the RA. To characterize AF propagation, offline analysis was conducted in a specialized software program (MATLAB 8.1; The MathWorks, Inc., Natick, MA, USA) as previously described [40, 43]. The identification of local activation times (ATs) and the determination of the fractionation index (FI) were based on a probabilistic algorithm [44] in a 60-s AF recording. The AT allowed for the determination of AF cycle length (AFCL) and maximal instantaneous time differences between neighboring electrodes (local electrical dissociation). Further ATs were used to reconstruct fibrillatory waves. The number of waves was normalized to the cycle length.

### Tissue Analysis

The anesthetized animals were sacrificed by exsanguination from a puncture in the left ventricle. The heart was quickly removed, rinsed from blood coagulants, and weighed. Biopsies were obtained from the LA and RA anterior/free wall (Supplementary Fig. S1). One large sample was subdivided into three smaller samples consisting of 1 cm × 1 cm dissections. Samples from each site were preserved in three different ways: (i) fixated in 10% formaldehyde for 24 h, followed by a temporary storage in alcohol; (ii) stored in RNAlater at − 20 ℃; and (iii) snap frozen in liquid nitrogen and stored at − 80 ℃ until further analysis.

### Quantification of Gene Expression of ANP, Col1a2, TGF-β, TNF-α, and IL-6

Tissue samples from LA and RA were processed for quantification of the expression of atrial natriuretic peptide (ANP), collagen type 1 alpha 2 chain (Col1a2), transforming growth factor beta (TGF-β), tumor necrosis factor alpha (TNF-α), and interleukin 6 (IL-6). Total RNA was extracted using peqGold TriFast (#30–2010; PeqLab) extraction reagent according to the manufacturer’s protocol, and genomic DNA impurities were removed by DNase treatment (PeqLab). Synthesis of complementary DNA (cDNA) was performed using the HighCap cDNA RT Kit (#43,690,616, Applied Biosystems). Quantitative real-time PCR was performed in a StepOnePlus thermocycler (Applied Biosystems) using TaqMan GenEx Mastermix (#4,369,016, Applied Biosystems). No template controls were used to monitor for contaminating amplifications. The ΔCt was used for statistical analysis and 2^−ΔΔCt^ for data presentation. Gene expression was normalized to glyceraldehyde 3-phosphate dehydrogenase (GAPDH). Probes used to amplify the transcripts were as follows: TGFB1 (Ec03468030_m1), COL1A2 (Ec03469522_m1), NPPA (Ec03468698_g1), TNF (Ec03467871_m1), IL-6 (Ec03468678_m1), and GAPDH (Ec03210916_gH).

### Quantification of Oxidative Stress Markers (SOD1, SOD2, PrxII, PrxIII, Prx-SO3, and Catalase Protein)

For the quantification of established markers of cardiac oxidative stress, standardized Western blotting was carried out. Tissue from the LA and RA was homogenized in homogenization buffer (5 mM EDTA, 25 mM NaF, 300 mM sucrose, 30 mM KH_2_PO_4_, pH 7.0), containing complete protease inhibitors (Roche Diagnostics; Cat. No.: 11873580001), PhosSTOP (Roche Diagnostics; Cat. No.: 04906845001), and 1 mM PMSF, and centrifuged at 16,000 g for 10 min. Fifty micrograms of protein was separated on 10% SDS-PAGE and electrophoretically transferred to nitrocellulose membranes (0.2 µm pore size, Bio-Rad, #1,620,112). Membranes were blocked in phosphate-buffered saline containing 5% nonfat dry milk for at least 120 min at room temperature and exposed to the following primary antibodies overnight: anti-superoxide dismutases 1 and 2 (SOD1 and SOD2, respectively), anti-catalase, and anti-peroxiredoxins 2 and 3 (Prx2 and Prx3, respectively). For antibody specifics, readers are referred to Supplementary Table S2. Antibody specificity for horse protein was tested using mouse LV proteins as positive control (Supplementary Fig. S1). Respective secondary antibodies (purchased from Sigma: anti-mouse: #A5278, anti-rabbit: #A6154) were incubated for 60 min at room temperature. Proteins were visualized by enhanced chemiluminescence according to the manufacturer’s protocol (#RPN2106, Amersham Pharmacia Biotech) and analyzed using the Fusion SL gel documentation system (PeqLab). Data are presented as intensity optical density.

### Immunohistochemical Staining for Quantification of Structural Remodeling and Fibroblasts

Samples from RA and LA free wall were processed for immunohistochemical staining to determine myocyte cell size, capillary density, fibroblast count, and the level of interstitial fibrosis, as described previously [45]. Here, anti-vimentin stains fibroblast/myofibroblasts, wheat germ agglutinin (WGA) labels glycoproteins in the extracellular matrix (ECM) and thereby also defines the cell membrane of the cardiomyocytes, and isolectin GS-IB4 is applied to identify endothelial cells. For further details on antibodies and concentrations, see Supplementary Table S2. Images were acquired using a Zeiss Axio Scan.Z1, a fluorescence microscope combined with AxioCam MRm at × 20 magnification using appropriate filters. Automated analysis of the images was performed using a custom-coded algorithm (JavaCyte) in ImageJ [45].

### Statistics

Statistical analysis was performed using GraphPad Prism 8 software (GraphPad Software, San Diego, CA, USA). Data was tested for normality using the Shapiro–Wilk test. All data are presented as mean and standard deviation (SD) unless otherwise stated. Unpaired *t* tests corrected for multiple testing using Tukey’s post hoc test were performed as well as mixed-effects analysis for repeated measures, followed by Sidák’s post hoc test. Nonparametric data was analyzed using the Mann–Whitney *U* test. A two-sided *p* value < 0.05 was considered statistically significant.

## Results

### Study Groups and Exercise Performance

**Fig. 2:**
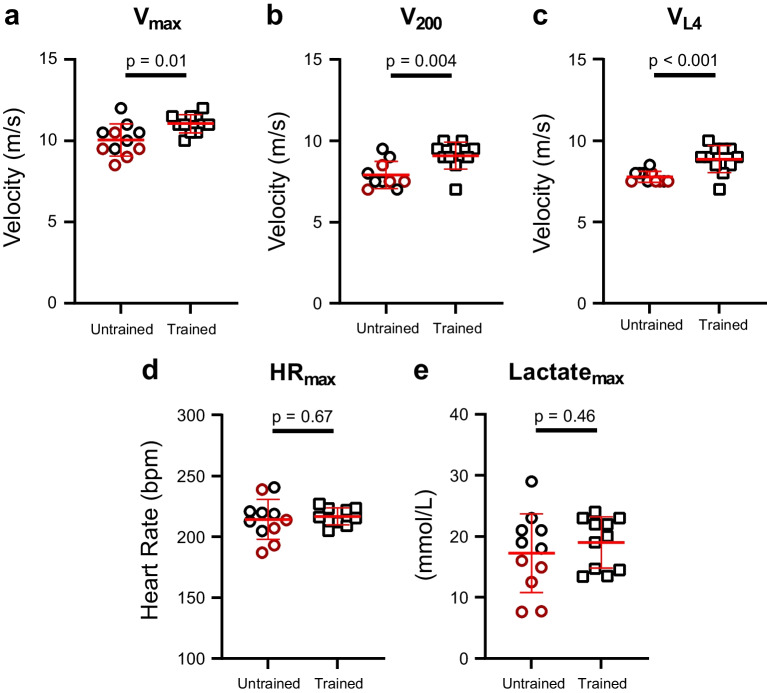
Standardized exercise test. The level of performance was based on **a** maximum velocity (*V*_max_), **b** velocity at a heart rate of 200 beats per minute (bpm) (*V*_200_), and **c** velocity at blood lactate 4 mmol/l (*V*_L4_) where trained horses performed significantly better in all parameters. To ensure equal exhaustion, maximum heart rate (HR_max_) and maximum lactate level (Lactate_Max_) were evaluated, revealing no difference between groups (**d** and **e**). Data is presented as mean ± standard deviation. Unpaired *t* test was applied. Completely untrained horses are presented as red circles

Of the 24 horses, one trained and one untrained horse were excluded, as they did not complete the protocol due to lameness and systemic disease (Supplementary Table S1). Trained horses performed significantly better on the treadmill (Fig. 2a–e).

### Gross Cardiac Changes

Trained horses had significantly larger hearts (mean weight/BW: 0.90 ± 0.05%, *n* = 9) compared to untrained animals (mean weight/BW: 0.81 ± 0.09%, *p* = 0.01, *n* = 7). Echocardiographic analysis revealed a significantly larger LV mass in trained animals but no difference in LA dimension or function (Table 1). When comparing LA variables to existing reference values [38], 2/11 trained horses had a LA_Diameter_ above the upper limit compared to the general population, as opposed to 0/11 in the untrained group. No correlation between LA size and AF duration was identified.

**Table 1:** Resting ECG and ECHO measurements

	Untrained (*n* = 11)	Trained (*n* = 11)	Effect size (95% CI)	*p* value
	Mean ± SD (95% CI)	Mean ± SD (95% CI)		
Electrocardiography				
HR (bpm)	28.8 ± 3 (27:30)	28.4 ± 2 (27:30)	− 0.4 (− 2.6:1.7)	0.67
P wave duration (ms)	141 ± 16 (130:151)	149 ± 14 (139:158)	7.7 (− 5.5:21.0)	0.24
PR interval (ms)	374 ± 45 (344:404)	423 ± 42 (395:451)	48.9 (10.5:87.4)	0.02*
QRS duration (ms)	118 ± 10 (111:125)	112 ± 16 (102:123)	− 5.6 (− 17.3:6.1)	0.33
QT interval (ms)	543 ± 47 (511:574)	542 ± 21 (528:556)	− 1.3 (− 33.6:31.1)	0.93
QTc interval (ms)	466 ± 46 (435:497)	462 ± 28 (443:480)	− 4.4 (− 38.1:29.4)	0.79
APC/20 h	0.0 (IQR: 0–3)	0.5 (IQR: 0–1.8)	0.5	0.97
2.AV blocks/20 h	6.0 (IQR: 0–110)	251.0 (IQR: 42–894)	245	0.06
Echocardiography				
LV_mass_ (% of BW) *n* = 10 + 11	0.56 ± 0.11 (0.49:0.64)	0.75 ± 0.13 (0.66:0.83)	0.2 (0.1:0.3)	0.003*
LA_Diameter_ (cm) *n* = 11 + 11	11.41 (IQR: 10.9–12.2)	11.83 (IQR: 11.4–12.53)	0.4	0.22
LA_Area_ (cm^2^) *n* = 11 + 11	80.17 (IQR: 76.6–90.2)	84.97 (IQR: 81.7–93.0)	4.8	0.12
LA-FAC_total_ (%) *n* = 8 + 9	36.15 ± 7.29 (30.1:42.2)	32.94 ± 7.05 (27.5:38.4)	− 3.2 (− 10.6:4.2)	0.37
LA-FAC_passive_ (%) *n* = 8 + 9	27.72 ± 7.60 (21.4:34.1)	23.76 ± 4.15 (20.6:27.0)	− 4.0 (− 10.2:2.3)	0.20
LA-FAC_active_ (%) *n* = 8 + 9	11.60 ± 4.93 (7.5:15.7)	11.90 ± 7.34 (6.3:17.5)	0.3 (− 6.3:6.8)	0.92

Comparison of ECG variables (HR, P wave duration, PR interval, QRS duration, QT interval, and corrected QT interval (QTc)) obtained at rest along with LV mass and LA size/function between trained and untrained horses. Left atrial function is based on the following equations: LA-FAC_total_ = (LA_Amax_ − LA_Amin_) / LA_Amax_ representing the overall atrial function, LA-FAC_passive_ = (LA_Amax_ − LA_Aa_) / LA_Amax_ representing the passive atrial emptying during early diastole and diastasis, and LA-FAC_active_ = (LA_Aa_ − LA_Amin_) / LA_Aa_ representing the active atrial contraction. Electrocardiographic data and LA functional values are presented as mean ± SD and 95% CI levels whereas the number of arrhythmias and LA dimension measurements are presented as median and IQR. The unparied *t* test and Mann–Whitney *U* test were applied. Effect size is based on the differences of the means. *p < 0.05

*HR* heart rate, *APC* atrial premature complex, *2.AV* second-degree atrioventricular, *LV* left ventricle, *BW* body weight, *LA* left atrium, *FAC* fractional area change, *CI* confidence level, *SD* standard deviation, *IQR* interquatile range

### Electrocardiography

Electrocardiographic results are presented in Table 1.

Four horses had less than 24 h of ECG recordings available due to noise or interruptions in the recordings, and one of these horses only had a total of 20 h available. Therefore, the assessment of second-degree AV block was based on a 20-h recording from each horse.

During the 20-h ECG recording, second-degree AV blocks occurred in 11/11 trained horses and in 8/11 untrained horses. A median of 251 s-degree AV blocks per 20 h was found in the trained horses (IQR: 42–894), as opposed to 6 per 20 h in untrained horses (IQR: 0–110, *p* = 0.06; Table 1).

### Electrophysiological Study (Conscious Horse)

Three horses (two trained and one untrained) did not complete the entire standing EP study due to S2-induced AF during aERP measurements that did not spontaneously cardiovert within an hour. These horses were able to undergo self-sustained AF and were allocated as having received one burst in order to be included in the AF inducibility data. As the baseline pacing at PCL 400 ms initiated small runs of AF, measurements of aERP at PCL 400 ms are missing from five horses (four trained and one untrained). There was no difference in RA aERP or rate dependency of the aERP between groups (Table 2).

**Table 2:** Atrial effective refractory periods in trained and untrained horses

PCL	Untrained	Trained	Effect size (95% CI)	Adjusted *p* value
	Mean ± SD (95% CI)	Mean ± SD (95% CI)		
aERP standing RA (ms)				
1000 ms	279 ± 49 (246:311)	282 ± 38 (254:311)	3.8 (− 36.6:44.2)	0.99
800 ms	276 ± 45 (245:308)	280 ± 39 (250:309)	3.1 (− 37.1:43.2)	0.99
500 ms	248 ± 29 (227:269)	254 ± 29 (232:276)	6.1 (− 21.8:34.1)	0.98
400 ms	228 ± 21 (212:244)	229 ± 21 (211:246)	0.6 (− 20.8:22.1)	> 0.99
aERP anesthesia RA (ms)				
1000 ms	266 ± 45 (234:298)	308 ± 48 (270:350)	42 (− 5.3:89.3)	0.22
800 ms	276 ± 29 (256:297)	307 ± 32 (273:341)	33.5 (− 1.8:68.8)	0.22
500 ms	268 ± 21 (250:286)	283 ± 12 (268:298)	14.9 (− 5.6:35.3)	0.25
400 ms	258 ± 13 (237:279)	254 ± 29 (208:299)	− 4.4 (− 47.1:38.3)	0.79
aERP anesthesia LA (ms)				
1000 ms	212 ± 48 (178:247)	251 ± 33 (229:274)	39.2 (7.3:71.06)	0.045*
800 ms	225 ± 38 (198:252)	262 ± 36 (237:286)	37.8 (6.0:69.7)	0.045*
500 ms	226 ± 27 (201:251)	263 ± 26 (245:282)	49.48 (16.5:83.0)	0.016*
400 ms	209 ± 29 (172:245)	242 ± 23 (184:299)	49.5 (9.8:89.1)	0.045*

Right atrial effective refractory period (ERP) in standing non-sedated horses and RA and LA aERPs in anesthetized trained and untrained horses at four different pacing cycle lengths. Data is presented as mean ± SD and 95% CI levels along with mean differences and adjusted *p* values. As the data contained missing values, a mixed-effects analysis for each parameter was applied. This analysis can, in cases of missing values, be interpreted as a repeated-measures analysis of variance (ANOVA). The analysis was followed by the Holm-Sidák post hoc analysis for multiple comparisons between trained and untrained horses at different pacing cycle lengths. Effect size is based on the differences of the predicted means constructed in the mixed-effects analysis. *p < 0.05

*RA* right atrium, *LA* left atrium, *PCL* pacing cycle length, *SD* standard deviation, *CI* confidence level

### Atrial Fibrillation Susceptibility and Stability

**Fig. 3:**
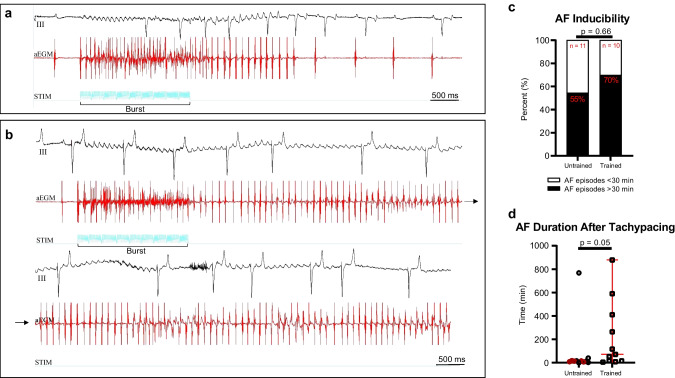
Atrial fibrillation inducibility and stability. Atrial fibrillation (AF) inducibility was evaluated in the standing non-sedated horses. Burst of 20 ms in a train of 200 stimuli was applied. **a** Representative ECG (lead III) and a right atrial electrogram tracing from an untrained horse where it was not possible to induce AF that could sustain > 30 min. **b** Representative tracing from a trained horse that only needed one burst to develop AF lasting > 30 min. **c** Seventy percent of trained horses went into AF lasting > 30 min compared to 55% in the untrained group (Fisher’s exact, not significant). After burst pacing, the horses were subjected to 20 h of tachypacing, where after spontaneous cardioversion to sinus rhythm was awaited. The trained horses maintained AF significantly longer than the untrained horses (**d**). The data in **d** is presented as median and quartiles. Completely untrained horses are presented as red circles. The Mann–Whitney *U* test was applied, and data is presented as median and interquartile range. aEGM, atrial electrogram; STIM, stimulation channel

Atrial fibrillation was sustained for > 30 min in 70% of the trained horses, whereas this applied for only 55% of the untrained horses. However, there was not any significant difference between the two groups (Fig. 3a–c). No difference in the duration of the non-sustained episodes (< 30 min) was found (median trained horses: 131 s, IQR: 87–462 s, compared to median untrained horses: 75 s, IQR: 63–733 s, *p* = 0.72).

After 20 h of tachypacing, the AF episode lasted significantly longer in trained horses (median: 71 min, IQR: 15–411 min) compared to untrained horses (median: 14 min, IQR: 7–18 min, *p* = 0.045; Fig. 3d).

### Electrophysiological Changes in the Anesthetized Horse

For RA, no differences in aERP meassurements were found between groups (Table 2) aligning with the results from the conscious measurements. In LA, however, aERP was prolonged in the trained horses compared to untrained at all pacing rates, remaining significant after correcting for multiple comparisons (*p* < 0.05 for all, Table 2).

### Electrophysiological Properties During AF

**Fig. 4:**
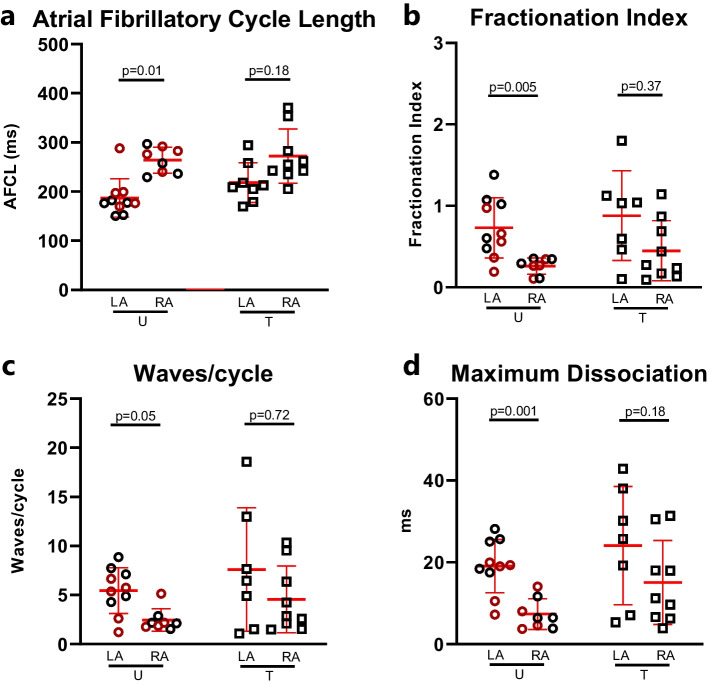
Atrial fibrillatio propagation pattern. AF complexity was addressed using HD contact mapping in an open-chest study. **a** Atrial fibrillatory cycle length, **b** fractionation index, **c** waves per cycle, and **d** maximum dissociation, a measure of the largest dissociation from an activation point. In the untrained group, all of these variables were significantly different between the right and left atria. This difference was attenuated in the trained group. Data is presented as mean ± standard deviation, and for statistical analysis, multiple unpaired Mann–Whitney (**a**–**c**) and *t* test (**d**) were performed followed by the Holm-Sidák post hoc analysis for correction. Completely untrained horses are presented as red circles. AFCL, atrial fibrillation cycle length; LA, left atrium; RA, right atrium; U, untrained; T, trained

The AFCL, FI, local dissociation, and waves/cycle were not different between trained and untrained horses (*p* > 0.05 for all). However, AFCL was significantly shorter and FI, local dissociation, and waves/cycle were significantly higher in the LA compared to those in the RA in the untrained group. This difference was not present in the the trained group (Fig. 4a–d).

### Molecular and Structural Remodeling

**Fig. 5:**
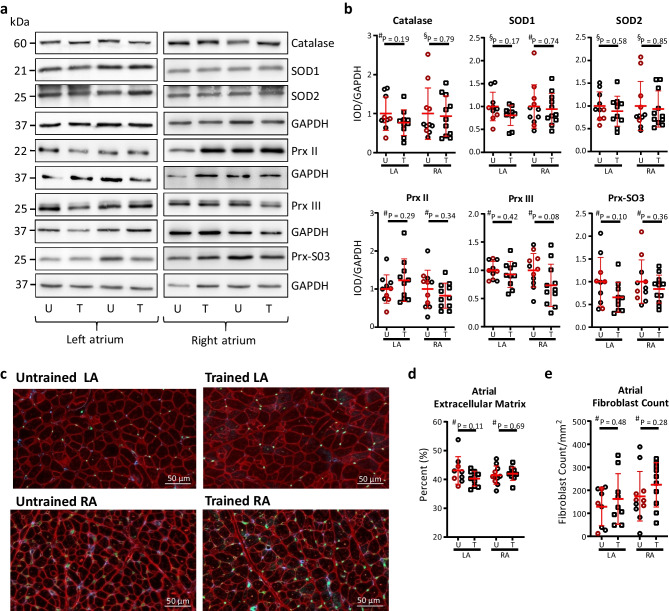
Inflammatory profile in untrained and trained horses. Western blot was performed to quantify the expression of prominent antioxidant enzymes (superoxide dismutases 1 and 2 (SOD1 and SOD2, respectively), peroxiredoxins (Prx2, Prx3, and Prx-SO3), and catalase with GAPDH as a housekeeping gene). No difference was found in the protein expression of either enzymes. **a** Representative blots of antioxidant enzymes in the left atrium (LA) and right atrium (RA) from two horses in each group. **b** Expression levels of enzymes. Triple immunofluorescent staining (representatives in **c**, scale bar 50 µm) was performed to quantify the presence of extracellular matrix and fibroblast, again showing no difference between groups (**d** and **e**). Data is presented as mean ± standard deviation. Red represents extracellular matrix, green marks endothelial cells, whereas blue marks fibroblasts. Completely untrained horses are presented as red circles. U, untrained; T, trained. ^#^Unpaired *t* tests, ^§^Mann–Whitney *U* test

No difference between groups was found in the ANP, Col1a2, TGFβ, TNF-α, and IL-6 gene expression (Supplementary Fig. S2). Furthermore, we did not detect any difference in SOD1, SOD2, PrxII + III, Prx-SO3, and catalase protein expression (Fig. 5a, b). No difference in either cell size, capillary density (Supplementary Fig. S3), fibroblast count, or the amount of ECM (Fig. 5c–e) was identified. Representatives of the triple staining are presented in Fig. 5c.

## Discussion

This study showed that AF sustainability was higher in horses subjected to long-term training for racing purposes, although they were not more susceptible to acute AF induction compared to their untrained counterparts. Trained horses exhibited longer aERP in the LA compared to untrained horses, which could indicate electrical remodeling; however, the induced AF propagation patterns did not differ between groups. Interestingly, our study provides no evidence supporting the presence of increased inflammation or structural remodeling as a result of long-term training. These findings are in contrast to the discoveries in rodents subjected to long-term intensive training [17–19].

### Athlete’s Heart

The trained horses in this study displayed typical characteristics of the athlete’s heart, including increased LV mass and prolonged PR interval, consistent with training-induced cardiac remodeling when compared to untrained and retired horses. In humans, atrial dilation is common for athletes [9]. We observed a slight increase in LA_Area_ and LA_Diameter_ in the trained horses compared to the untrained ones; however, this was not significant and we did not identify cellular hypertrophy in any of the atria. Still, approximately 18% of the trained horses presented with atrial enlargement when compared to established reference values for 500-kg horses [38]. However, the reference values are based on measurements from warmblood horses and caution should be made when interpreting this comparison, as breed differences may exist. In humans, LA enlargement is present in approximately 20% of athletes, where it is most pronounced in endurance athletes and athletes combining static and dynamic exercises, i.e., cyclists and rowers [9, 46, 47]. Furthermore, the increased risk of AF in athletes is the most noticeable in high-intensity sports practitioners [3, 48, 49]. Nonetheless, the direct association between exercise-induced increased LA size and AF is very low [46] and we did not identify any association between LA size and the stability of AF, suggesting that atrial dilation in athletes may not contribute substantially to the development of an arrhythmogenic substrate. Importantly, however, the power to detect very subtle or non-ubiquitous changes in LA size may be limited when only a small number of animals were included, and the possibility of type 2 errors needs to be taken into consideration.

Besides an increase in LA size, increased vagal activity was essential for increasing AF susceptibility in a rat model of exercise-induced AF [18]. Guasch et al. [18] showed an increased baroreceptor and cholinergic sensitivity in their trained group, in which atropine terminated AF, suggesting that vagal enhancement was crucial for increasing AF susceptibility in their model. Although recently challenged [50, 51], it has generally been accepted that vagal tone is enhanced in athletes and that this enhancement is pro-arrhythmic [52–55]. We did not look directly into autonomic imbalances in our model, and the role of vagal tone and AF remains unexplored in horses. We observed an increased PR interval and more second-degree AV blocks, which can be interpreted as an indicator of increased parasympathetic activity. We previously showed that intrinsic remodeling of the AV node—and not increased vagal activity—explained the slowed AV conduction seen in the trained horses [30]. Nevertheless, as training is not a prerequisite for some horses to exhibit a second-degree AV block, which potentially is related to differences in the expression of muscarinic receptors in the AV node (unpublished data), it seems that the burden of second-degree AV block may come down to a multitude of factors and the high burden in trained horses may be a result of many mechanisms, including increased vagal tone, intrinsic remodeling, and even other unknown mediators.

### AF Inducibility and Stability

AF was not more readily induced in the trained cohort, suggesting that the level of training-induced remodeling was not sufficient to drive AF alone. However, combined with acute electrical remodeling induced by 20 h of rapid pacing, training increased the stability of AF. In a previous acute AF study in horses, electrical remodeling occurred already after 4 h of AF [56], making it reasonable to expect an electrical remodeling in our tachypaced horses. In rats, an altered gene expression of different ion channels was already present within 8 h of tachypacing, where increased mRNA levels of Kv1.5, underlying *I*_Kur_, most likely resulted in shortening of the action potential [57]. Furthermore, studies have shown that 24 h of rapid pacing in dogs induced a shortened action potential duration in atrial myocytes due to a reduction of the transient outward current, *I*_to_, and the L-type Ca^2+^ current caused by transcriptional downregulation of ion channels mediating these currents induced by Ca^2+^ overload [58, 59]. Interestingly, studies in mice showed that training improved myocyte contractility and Ca^2+^ handling via increasing calcium/calmodulin-dependent kinase II delta (CAMKII) phosphorylation [60]. An increase in CAMKII phosphorylation will, in turn, increase the phosphorylation of phospholamban, removing its inhibitory effect on sarcoplasmic/endoplasmic reticulum Ca^2+^ ATPase 2a, which will increase the reuptake of Ca^2+^ into the sarcoplasmic reticulum. This induces increased Ca^2+^ sensitivity in the trained animals [60], and as tachypacing can lead to Ca^2+^ overload [58, 59], we speculate that this combination may be involved in the increased AF stability in the trained horses after 20 h of tachypacing. In general, tachycardia has been shown to lead to alterations in calcium handling, leading to an increased number of calcium sparks, as well as early and/or delayed after depolarization that can both trigger and sustain AF [61]. A central etiology of AF in humans is pulmonary vein (PV) firing, which may also be calcium dependent. Interestingly, PV firing frequency has been shown to increase both in periods of tachycardia [62] and in rapid changes in autonomic tone [63, 64]. Furthermore, runs of AF itself increase PV firing and the combined training-induced changes and transient tachypacing could mimic clinical cases of trained individuals undergoing different tachycardia episodes induced by exercise bouts, PV firing, paroxysmal AF, or tachycardia prompted by other means. Calcium handling properties are central in AF pathogenesis and are very sensitive to small physiologic changes. For further insights in calcium handling and AF pathogenesis, the readers are referred to an excellent review by Denham et al. [61].

In general, short episodes of induced AF shorten the action potential duration and the aERP in animal models, including horses [56], which theoretically favors more reentry circuits and increases AF susceptibility [65]. However, depending on studies, it has been observed that humans with structural heart diseases and AF have prolonged aERP [66], which also applied for patients suffering from paroxysmal AF [67], questioning the pathophysiologic consequences of long vs. short atrial refractoriness. We observed an increased aERP in the LA in trained horses, and similar to our findings, the action potential duration of LA cardiomyocytes in swim-exercised mice was prolonged, despite being more susceptible to AF [19]. As such, it seems that training induces electrical atrial remodeling in the LA, but it is not possible to conclude whether this led to the increased AF stability in our study. It has to be considered that the spatial dispersion in atrial refractoriness would have been another measure of interest as an increase in vagal tone would enhance the dispersion in refractoriness and this would indeed be pro-arrhythmic [68].

### Left and Right Atrial Heterogeneity and AF Stability

Differences between RA and LA in terms of AF stability and complexity were found, and the AF complexity variables were higher in LA, which is in agreement with other studies [40, 69, 70]. The difference in AF complexity seen between RA and LA in the untrained horses was less pronounced in the trained horses, where the data variability, in general, was higher compared to the untrained horses. In fact, the SD was more than double in all the epicardial mapping data from the RA in trained horses compared to RA data from untrained, possibly explaining the lack of statistical difference in the trained group. Local electrical heterogeneity is believed to be a driver of AF. However, when it comes to a difference between RA and LA, it might be that the slightly increased AF complexity in the RA and a less pronounced atrial gradient are more favorable to maintain AF, as both atria would add new wave fronts to the contralateral atrium instead of just having one following the other. Similar mechanisms have previously been shown in horses treated with a small conductance Ca^2+^-activated K^+^ (SK) channel blocker, where the blocking effect had different impacts on LA and RA [40]. In that study, AF was more complex in the LA at baseline, but the SK blocker did not alter AF propagation in the LA. Instead, the SK blocker increased the stability of AF by increasing the complexity of the arrhythmia in the RA and thereby prevented termination of AF, similar to the effect of training in our horses.

### Structural Remodeling

Exercise-induced structural remodeling has been shown in several rodent models [17, 18, 71], where especially a marked increase in atrial fibrosis has been identified and considered to be a possible mechanism for AF development in athletes [53]. In our study, we did not observe increased ECM, nor did we identify an increased number of fibroblasts or increased expression of pro-inflammatory and oxidative stress–related markers. Hence, no sign of increased inflammation was present in the trained horses, and yet, they maintained AF longer. Although we did not detect a difference in total ECM in the RA/LA within groups or between groups, there may still be a difference in the various types of collagens or collagen precursors. In the myocardium, types I and III are the major components of collagen [72]. However, the WGA staining used in our study does not allow for differentiation between collagen types. The amount of fibrosis in our analysis is based on a staining that represents the ECM, but as ECM does not only consist of collagen, other ECM components may attenuate changes specifically related to collagen amount and composition. We detected a significant difference when we compare the level of Col1a2 between LA and RA in trained horses, indicating some signs of remodeling.

The horses in our study had been subjected to three or more years of training 3–4 times per week, which is standard in the trotting industry, in order for the horses to compete at high levels. Exercise models in animals are normally based on relatively short high-intensity training, such as rats vigorously trained for 16 weeks [17, 18, 71]. It is possible that these training regimens, which are against natural behavior for rodents, lead to another phenotype that elicits a stress-related impact which could play a pivotal role, e.g., influencing the development of fibrosis. Our findings concerning fibrosis align with relatively large studies in human athletes, which did not detect any signs of cardiac fibrosis using cardiac magnetic resonance imaging [22, 24, 73]. Other studies, including both veteran and young elite athletes, did find minor fibrotic changes in the ventricles that differed from the general population [23, 25, 26], but no atrial findings were reported. As such, major changes in atrial interstitial composition may not be expected in human or horse athletes, as opposed to rats undergoing forced high-intensity training. However, as half of the untrained horses in our study had undergone some training very early in life, this could perhaps explain the lack of difference between the two groups. Despite the fact that the LV size had returned to normal in the retired horses, some structural changes may not have reversed. One of the retired horses did maintain the tachypacing-induced AF much longer than the remaining control group (> 12 h). This horse did not differ in terms of electrophysiology and in atrial size. Furthermore, this horse did not have an increased inflammatory response or fibrosis in general, compared to the horses that had never trained. Whether increased fibrosis is present in human athletes remains debated, as certain biomarkers of structural remodeling, such as tissue inhibitors of matrix metalloproteinase type I (TIMP-1), carboxy-terminal propeptide of collagen type I (PICP), carboxy-terminal telopeptide of collagen type I (CITP), and tumorigenicity 2 (sST-2), have been found to be elevated in athletes in some studies [26, 74], whereas other were not able to detect any increase in inflammatory markers [75]. Furthermore, most of these biomarkers are transiently elevated after exercise and it is impossible to identify the exact origin of many circulating plasmatic markers, as they may arise from extra-myocardial regions as well.

## Limitations

This study investigated the effects of training on atrial remodeling in racehorses, and therefore, translation to humans should be undertaken with caution. The most important limitation to the study is that our model does not have spontaneously occurring AF. In fact, most endurance athletes and trained horses do not develop spontaneous AF. Therefore, the structural or electrical prerequisites for AF to occur spontaneously may be less common and different than changes observed in the general population of trained individuals, where the majority would never develop AF. This may also explain the lack of differences seen in this study.

Another important limitation is the fact that training intensity of the trained cohort was not logged in detail, and it is posssible that the horses may have been trained at different intensities and durations. Additionally, it is difficult to find Standardbred racehorses that have never been trained, at least if age-matched controls are desired. We decided to include horses that had been subjected to training early in life, which could have potentially induced irreversible cardiac remodeling. This may raise the question as to whether these two groups of horses were distinct enough in order to detect the atrial remodeling that may be present in extremely trained individuals. However, as the general population is hardly completely inactive throughout their entire lifetime, this study may, in fact, resonably represent the human athlete and their untrained counterpart. Both mares and geldings were included, which may be a limitiation, as sex may influence ECG parameters. The very large size of the horse atrium is problematic, as we could not map the entire atrium. Likewise, all the tissue analyses had only been conducted on a limited amount of tissue. This may have prevented us from identifying local changes in the atria. Furthermore, analysis of differential cardiac gene expression patterns and altered protein concentrations is highly limited by the availablity of horse-specific PCR probes and antibodies.

## Conclusion

Trained horses presented with a better substrate for sustaining tachypacing-induced AF compared to untrained control horses. This substrate was not associated with any signs of increased inflammation, fibrosis, or atrial cellular hypertrophy. However, trained horses showed longer LA aERP and the AF propagation patterns in the RA and LA were similar in complexity in the trained group, whereas a difference between RA and LA in the untrained horses was identified. Training increases the propensity of AF without substantial effects on the common pathophysiological etiology of AF. More studies are needed to identify other changes in the underlying mechanism of AF in the athlete’s heart.

### Supplementary Information

Below is the link to the electronic supplementary material.

Supplementary file1 (PDF 883 KB)

Supplementary file2 (PDF 444 KB)

## Abbreviations

aERP: Atrial effective refractory period
AF: Atrial fibrillation
AFCL: Atrial fibrillation cycle length
APC: Atrial premature complex
AT: Activation time
AV: Atrioventricular
BW: Body weight
ECG: Electrocardiogram
ECHO: Echocardiogram
EP: Electrophysiological
FI: Fractionation index
HR: Heart rate
LA: Left atrial/atrium
LV: Left ventricular/ventricle
PA: Pulmonary artery
RA: Right atrium
