# Supplementary material for: Long-Term Training Increases Atrial Fibrillation Sustainability in Standardbred Racehorses
Source: J Cardiovasc Transl Res. 2023 Apr 4;16(5):1205–19. doi: 10.1007/s12265-023-10378-6 (PMC10615936; doi:10.1007/s12265-023-10378-6)
Supplement: Supplementary file 1 — Supplementary file1 (PDF 883 KB) [file 12265_2023_10378_MOESM1_ESM.pdf]

Supplementary material

Table S1. Horses, training status, sex, age and weight.
The training status of the horses were based on previous owner communication and by extracting racing activity from the Danish race registry (www.trav.dk)

**Horse ID**	**Training status** **(**years since training ceased**)**	**Sex**	**Age** (years)	**Weight** (kg)
**EX1**	Trained	Gelding	9	565
**EX2**	Untrained (2 years)	Gelding	7	460
**EX3**	Trained	Mare	5	465
**EX4**	Trained	Gelding	10	404
**EX5**	Untrained (4 years)	Mare	9	515
**EX6**	Untrained (4.3 years)	Mare	10	490
**EX7**	Trained	Gelding	9	520
**EX8**	Untrained/never trained	Mare	6	462
**EX9**	Untrained/never trained	Mare	6	528
**EX10**	Untrained (4.9 years)	Mare	9	500
**EX11**	Untrained/never trained (Excluded)	Mare	7	548
**EX12**	Trained	Mare	5	490
**EX13**	Trained	Mare	7	441
**EX14**	Untrained (>4.1 years)	Mare	9	415
**EX15**	Untrained (never trained)	Gelding	4	560
**EX16**	Trained	Mare	5	486
**EX17**	Untrained/never trained	Mare	5	534
**EX18**	Trained	Mare	7	462
**EX19**	Trained	Gelding	5	470
**EX20**	Trained	Gelding	6	504
**EX21**	Trained (Excluded)	Mare	7	495
**EX22**	Untrained/never trained	Gelding	5	447
**EX23**	Trained	Gelding	8	529
**EX24**	Untrained (>4.1 years)	Mare	8	554

At arrival, the horses underwent a thorough clinical examination, including standard blood hematology and biochemical analysis along with a lameness examination. After given two days to accustom to the new surroundings a 24-hour Holter electrocardiographic (ECG) (KRUTECH Televet, Kruuse A/S, Maarslev, Denmark) recording was obtained. A standardized echocardiographic (ECHO) (2-D, M-mode and color flow Doppler using a portable ultrasound unit (Vivid IQ, GE Healthcare, Horten, Norway) with a phased array transducer (3S Phased Array transducer, GE Healthcare)) examination was also performed. Only horses with no signs of cardiovascular disease were included, however trivial to mild valvular regurgitations were accepted. Lameness <grade 3 on the American Association of Equine Practitioners lameness grading scale was accepted.

Table S2. Antibodies used for Western blot and immuohistochemical triplestaining

Antibody	Target	Concentration
anti-superoxide dismutase-1 (SOD1; Santa Cruz Biotechnology, sc-11407)	superoxide dismutase-1	1:1000
anti-superoxide dismutase 2 (SOD2; Santa Cruz Biotechnology, sc-30080)	superoxide dismutase 2	1:1000
anti-Catalase (Cell Signaling Technology, #14097)	Catalase	1:1000
anti-peroxiredoxin 2 (Prdx-2; abcam #ab50862)	peroxiredoxin 2	1:1000
anti-peroxiredoxin 3 (Prdx3, abcam, #ab73349),	peroxiredoxin 3	1:1000
anti-peroxiredoxin-SO3 (Prdx-SO3; Abfrontier, LF-PA0004)	peroxiredoxin-SO3	1:1000
Anti-GAPDH (Millipore, #MAB374)	GAPDH	1:2500
Anti-vimentin (Rabbit Monoclonal to vimentin, Abcam	Fibroblasts/myofibroblast	1:150
Secondary fluorescein-labelled anti-vimentin secondary antibody (Goat Anti-Rabbit IgG Cross Absorbed, Alexa Flour 405, Thermo Fisher)	Anti-vimentin primary antibody	1:200
WGA (Alexa Flour 594 conjugate, ThermoFisher)	Extracellular matrix	1:200
GS-IB4 (Isolectin GS-IB4, Alexa Flour 488 conjugate, ThermoFisher)	Capillaries	1:200

Figure S1. Western blots showing AB specificity

Antibody specificity and proper antibody dilutions for horse protein was tested using protein lysates derived from either right ventricular (RV) or right atrial (RA) horse tissue. As positive control, proteins from left ventricular mouse tissue were used. For each Western blot, 50 µg of protein was separated on a 10% SDS-PAGE and electrophoretically transferred to nitrocellulose membranes as described in the method section. All primary antibodies were diluted 1:1000 in phosphate-buffered saline containing 5% nonfat dry milk, except for GAPDH which was diluted 1:2500. Antibodies incubated over night at +4°C on an overhead shaker. Respective secondary antibodies (diluted 1:10000) were incubated for 60 min at room temperature and proteins were visualized as described in the method section. M = marker; SOD = superoxide dismutase; LV = left ventricle, RV = right ventricle; RA = right atrium

GAPDH (Millipore #MAB 374)
Dilution: 1/2500

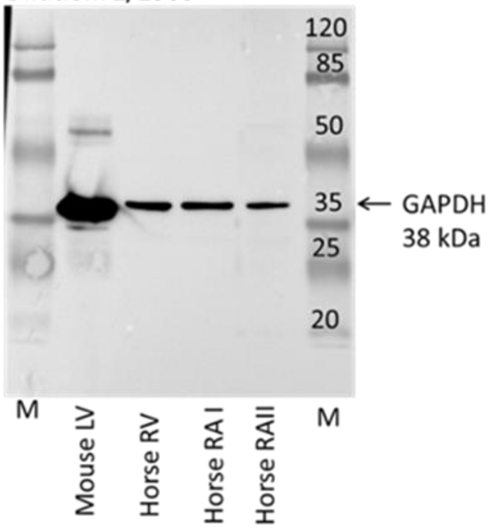

SOD1 (Santa Cruz #sc-11407)
Dilution:1/1000

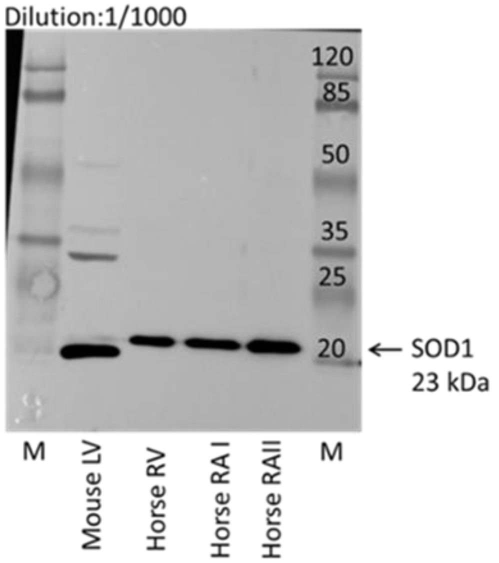

Catalase Cell Signaling #14097
Dilution: 1/1000

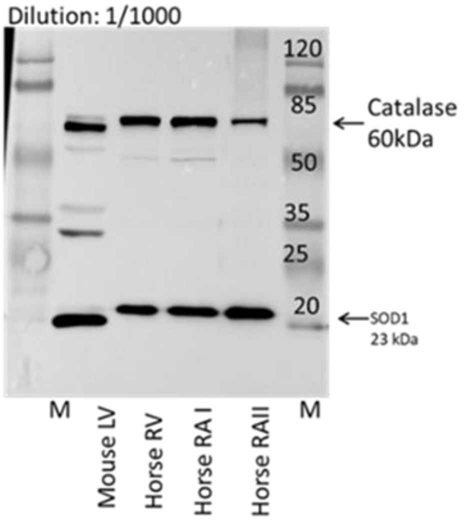

Prdx2 (abcam #ab50862)
Dilution: 1/1000

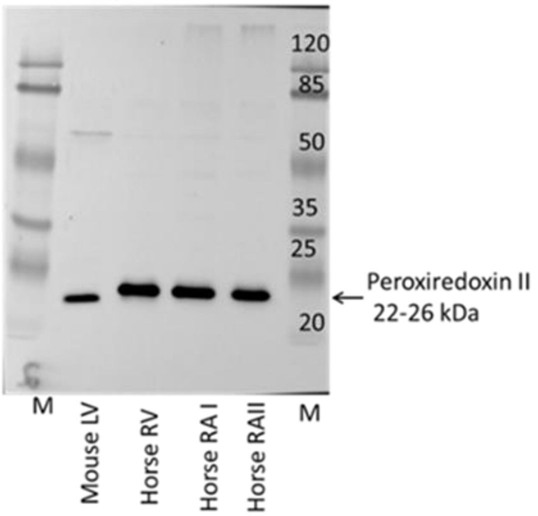

Antibodies diluted 1/1000

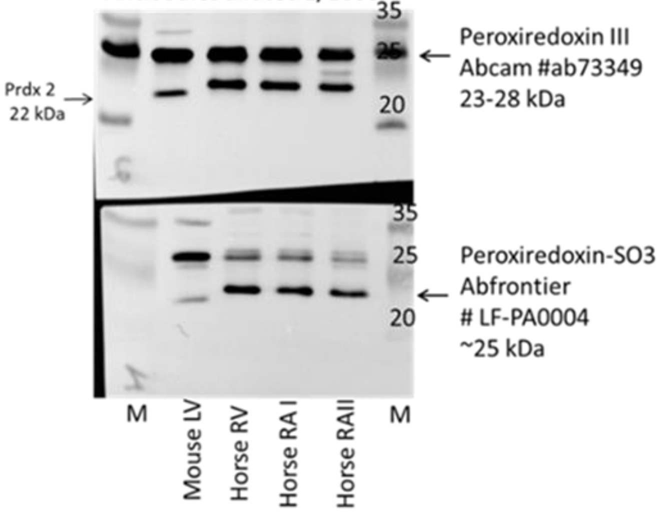

SOD2 (Santa Cruz #sc-30080)
Dilution: 1/1000

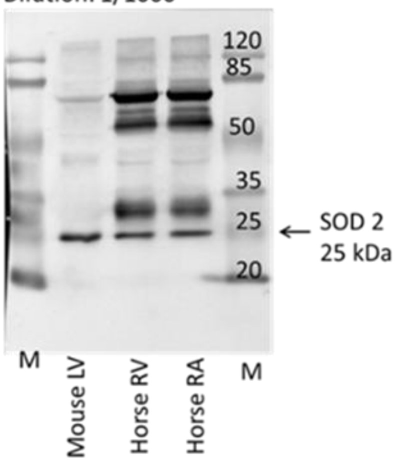

Figure S2. Gene expression of inflammatory markers

Expression profile of pro-hypertrophic atrial natriuretic peptide (ANP), pro-fibrotic collagen 1a2 (Col1a2) and transforming growth factor beta (TGFβ) as well as inflammatory tumor marker genes necrosis factor alpha (TNFα) and interleukin 6 (IL6) were analyzed in left- (LA) and right atrial (RA) tissue (n=10-11 per group). Glyceraldehyd-3-Phosphate-Dehydrogenase (GAPDH) served as housekeeping gene. Data is shown as mean ± standard deviation. # = Unpaired t-tests was applied for data with normal distribution. § = Nonparametric data was analyzed using Mann-Whitney U-test. U = Untrained, T = Trained.

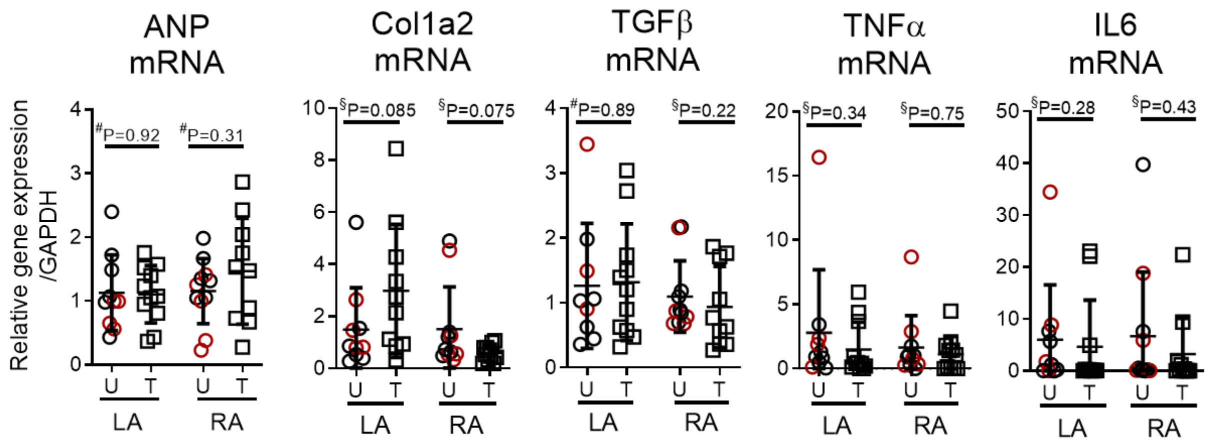

Figure S3. Immunohistcochemical triplestaining

Samples from right (RA) and left atrial (LA) free wall were processed for immunohistochemical staining to determine myocyte cell size and capillary density. In this, anti-vimentin stains fibroblast/myofibroblasts (data presented in main text), Wheat Germ Agglutinin (WGA) binds to extracellular matrix and defines the cell membrane of the cardiomyocytes and lastly isolectin GS-IB4 is applied to identify endothelial cells. Images were acquired using a Zeiss AxioScanner Z1, a fluorescence microscope combined with Axiocam MRm at 20x magnification using appropriate filters. Automated analysis of the images were performed using a custom-coded algorithm (Java Cyte) in ImageJ. Data are presented as mean ± standard deviation

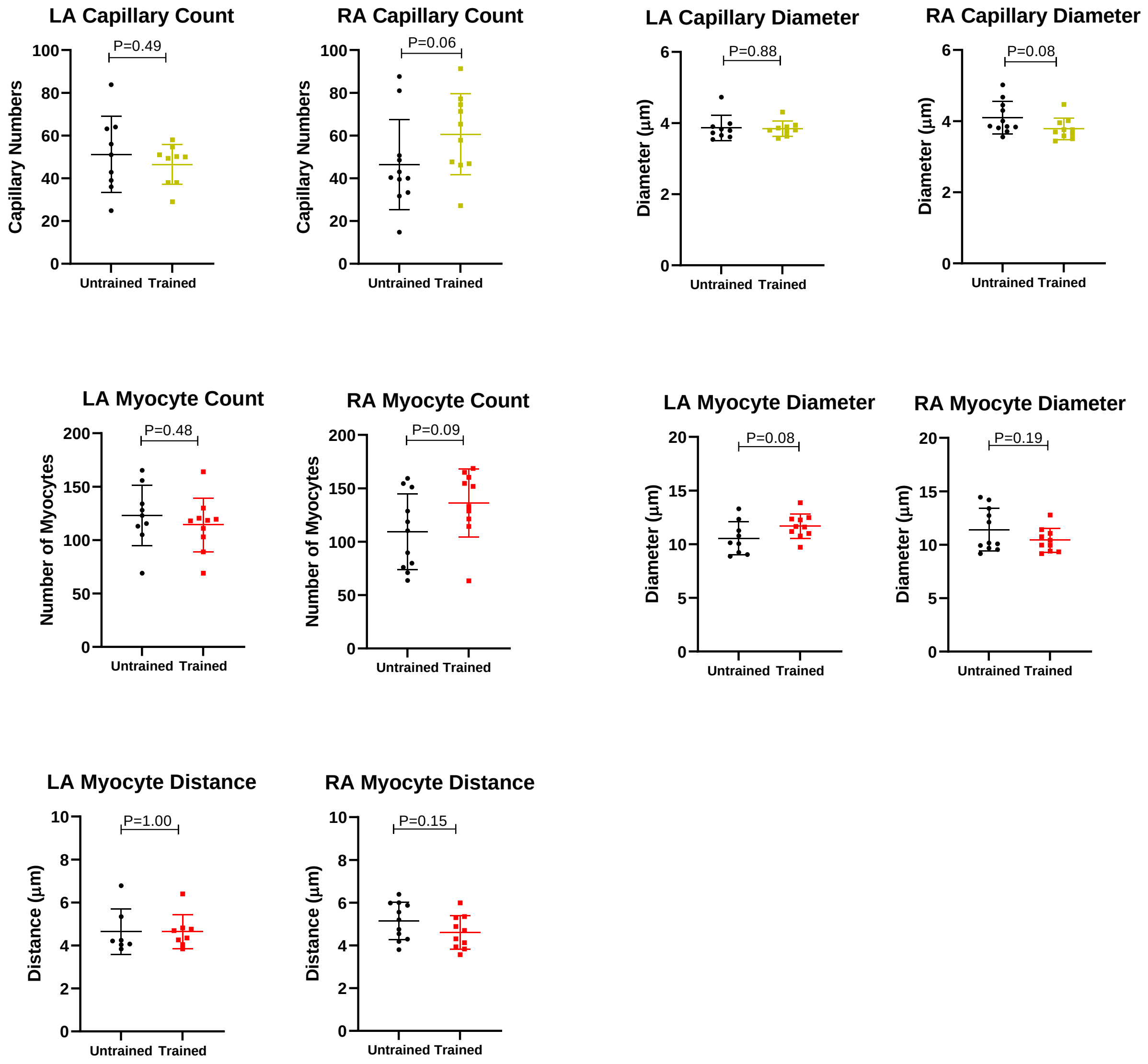

1 Fenner, M. F. *et al.* Inhibition of Small-Conductance Calcium-Activated Potassium Current (IK,Ca) Leads to Differential Atrial Electrophysiological Effects in a Horse Model of Persistent Atrial Fibrillation. *Frontiers in Physiology* **12**, doi:10.3389/fphys.2021.614483 (2021).

2 McGurrin, M. K. J., Physick-Sheard, P. W. & Kenney, D. G. How to perform transvenous electrical cardioversion in horses with atrial fibrillation. *Journal of Veterinary Cardiology* **7**, 109-119, doi:https://doi.org/10.1016/j.jvc.2005.09.001 (2005).

3 Adler, D. M. T. *et al.* Thoracotomy and Pericardiotomy for Access to the Heart in Horses: Surgical Procedure and Effects on Anesthetic Variables. *Journal of Equine Veterinary Science* **96**, 103315, doi:https://doi.org/10.1016/j.jevs.2020.103315 (2021).
