# Supplementary material for: Long-Term Training Increases Atrial Fibrillation Sustainability in Standardbred Racehorses
Source: J Cardiovasc Transl Res. 2023 Apr 4;16(5):1205–19. doi: 10.1007/s12265-023-10378-6 (PMC10615936; doi:10.1007/s12265-023-10378-6)
Supplement: Supplementary file 2 — Supplementary file2 (PDF 444 KB) [file 12265_2023_10378_MOESM2_ESM.pdf]

Figure S1. Biopsies of left and right atrium

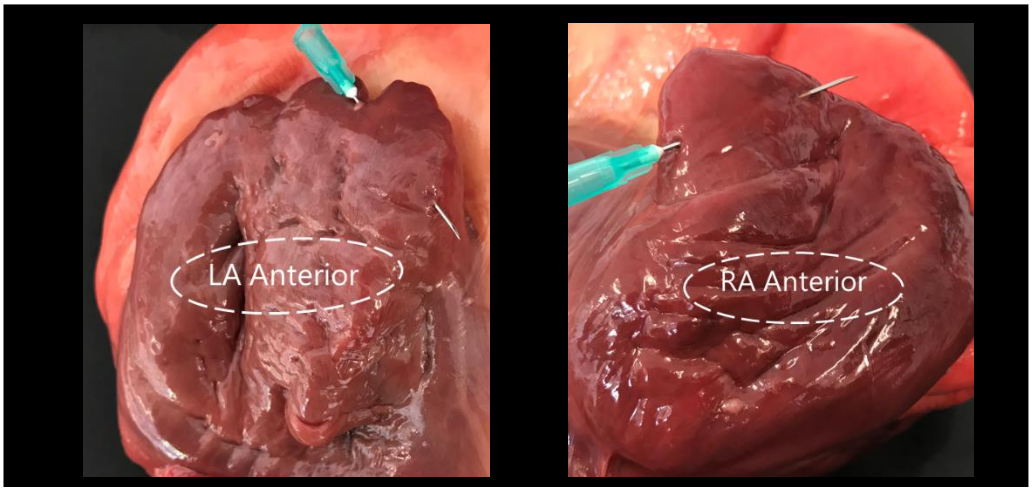

Figure 1: Left atrium (LA) seen from the above and right atrium (RA) seen from the above. One sample was taken from both atria and subdivided into three smaller samples of approximately 1x1 cm.
